# Progression of Renal Insufficiency in Patients with Essential Hypertension Treated with Renin Angiotensin Aldosterone System Blockers: An Electrocardiographic Correlation

**DOI:** 10.3390/diseases5040033

**Published:** 2017-12-08

**Authors:** Luis Rodriguez-Padial, Finn Akerström, María G. Barderas, Fernando Vivanco, Miguel A. Arias, Julian Segura, Luis M. Ruilope

**Affiliations:** 1 Department of Cardiology, Hospital Virgen de la Salud, 45005 Toledo, Spain; finnakerstrom@gmail.com (F.A.); maapalomares@secardiologia.es (M.A.A.); 2 Department of Vascular Physiopathology, Hospital Nacional de Paraplejicos, 45005 Toledo, Spain; marucitos@hotmail.com; 3 Department of Imunology, Fundacion Jimenez Diaz, UAM, 28220 Madrid, Spain; fvivanco@gmail.com; 4 Hypertension Unit, Instituto de Investigación imas12, Hospital Universitario 12 de Octubre, 28220 Madrid, Spain; jsegura@gmail.com (J.S.); lmruilope@gmail.com (L.M.R.)

**Keywords:** hypertension, ECG, renal function, microalbuminuria

## Abstract

Background: There is a frequent association between renal insufficiency and cardiovascular disease in patients with essential hypertension (HTN). The aim of this study was to analyze the relationship between ECG parameters and the progress of renal damage in patients with treated HTN. Methods: 109 patients with HTN had their microalbuminuria monitored over a 3-year time frame. During the last 3 months of follow-up, an ECG was recorded. Patients were divided into 3 groups according to the deterioration of their renal function: normoalbuminuria during the study period (normo–normo; *n* = 51); normoalbuminuria developing microalbuminuria (normo–micro; *n* = 29); and microalbuminuria at baseline (micro–micro; *n* = 29). Results: There were no differences in presence of left ventricular hypertrophy between the 3 groups. RV6/RV5 >1 was observed more frequently as renal function declined (*p* = 0.025). The 12-lead QRS-complex voltage-duration product was significantly increased in patients without microalbuminuria at baseline who went on to develop microalbuminuria (*p* = 0.006). Patients who developed microalbuminuria during follow-up, with positive Cornell voltage criteria, showed a lesser degree of progression of microalbuminuria when compared with the rest of the subgroups (*p* = 0.044). Furthermore, patients with microalbuminuria at baseline treated with angiotensin receptor blockers and diuretics, and positive Cornell voltage criteria, showed a higher degree of microalbuminuria compared to those with negative Cornell voltage criteria (*p* = 0.016). Conclusions: In patients with HTN, we identified some ECG parameters, which predict renal disease progression in patients with HTN, which may permit the identification of patients who are at risk of renal disease progression, despite optimal antihypertensive pharmacotherapy.

## 1. Introduction

The cardiorenal syndrome exemplifies the close relationship between heart and kidney [1]. Essential hypertension (HTN) is the result of a complex pathophysiological process with an associated elevated risk of developing cardiovascular [2] and renal disease [3]. HTN produces left ventricular hypertrophy (LVH) and dysfunction, which leads to increased cardiovascular morbidity and mortality [4]. The renal damage caused by HTN results in a progressive deterioration of glomerular filtration rate, often associated with microalbuminuria or proteinuria [2]. Furthermore, HTN seems to have a more deleterious effect on the heart when renal insufficiency (RI) is present [5], and we have recently described some electrocardiographic (ECG) parameters predictive of RI in individuals aged >65 years [6]. The renin–angiotensin–aldosterone system (RAAS), which plays an essential role in renal and cardiac damage could, amongst other mechanisms, represent one of the pathological processes that explain organ damage associated with HTN. 

Medical therapies like RAAS inhibitors (angiotensin converter enzyme inhibitors (ACEI) or angiotensin II receptor blockers (ARB)) have been demonstrated to reduce LVH to a greater degree than other antihypertensive therapies [7,8], and improve the prognosis in patients with heart failure. In the same way, the use of RAAS blockers in patients with diabetes mellitus slows down the deterioration of renal function [9]. Nevertheless, a significant proportion of patients continue the decline of renal function despite optimal medical therapy characterized by the increment or de novo microalbuminuria, suggesting a certain degree of resistance to RAAS blockers in some patients [10,11]. Our group have, through proteomic studies, identified some markers of renal damage (cerulopasmin, haptoglobin, and alpha-1-acid glycoprotein) [12], and we are currently focusing on the discovery of new risk markers that may allow for the identification of patients with a degree of resistance to RAAS blockage, since they represent individuals at risk of developing cardiorenal disease. 

The ECG represents an easy, rapid, economical and readily available test, which is frequently performed in patients with HTN. Therefore, the objective of this study was to analyze the relationship between different ECG parameters, and the progress of renal damage in patients with HTN receiving RAAS blockers, which could provide a tool for following the clinical evolution of these high risk patients.

## 2. Material and Methods

Patients: One hundred and nine patients with HTN with and without microalbuminuria (*n* = 31 patients with diabetes mellitus) were enrolled between January and June in 2012 at a specialized HTN clinic, with the aim to evaluate the progression of albuminuria during over a 3-year time frame. An ECG was recorded in all patients during the last 3 months of the study. During the study period, the patients had received RAAS blockers (ACEI or ARB) aimed at blood pressure (BP) control and reduction in renal disease progression characterized by the development of microalbuminuria. 

Patients were divided into three groups according to the deterioration of their renal function: those with normoalbuminuria during the study period (normo–normo) (*n* = 51), those with normoalbuminuria who developed microalbuminuria during the study period (normo–micro) (*n* = 29) and those with microalbuminuria at baseline and during the study period (micro–micro) (*n* = 29). 

The study was carried out according to the Helsinki declaration, and had previously been approved by the local ethical committee (protocol number: PI11/02432, approved on the 3 November 2011; Hospital 12 de Octubre, Madrid, Spain).

Electrocardiogram: The study group underwent a 12-lead ECG recording according to standard techniques in a prone and upright position using a MAC 1200 ST ECG recorder (GE Medical Systems) during the last 3 months of the study period. Digital recordings of the ECG (XML format) were stored in a database (GE Cardiosoft database v6.5, GE Healthcare, Chicago, IL, USA) for posterior analysis with the Electropres platform according to the Hannover ECG System(HES)^®^ criteria [13,14].

Electropres is an on-line system designed for the early detection of LVH by the ECG, using the HES^®^ for ECG measurement and interpretation. This software, approved by the FDA, has been shown to have a high precision in ECG interpretation [9], and has been validated in several clinical studies [15,16]. Voltage, duration, and area of all waves of the QRS complex were measured by the system, and several LVH criteria were calculated (Table 1).

Microalbuminuria: Urinary albumin excretion was measured by turbidimetry according to current recommended standards, and was reported as the albumin-to-creatinine ratio in milligrams per gram creatinine. Two morning urine samples were obtained from every patient and the average of the two was considered as the value of albuminuria. Patients were classified as normoalbuminuria (<30 mg/g), microalbuminuria (30–300 mg/g), and macroalbuminuria (>300 mg/g) [17]. 

Statistical analysis: Continuous and qualitative variables were expressed as mean ± standard deviation and percentage, respectively. Comparison between different groups according to age and QRS complex duration were carried out using the Student’s *t*-test or ANOVA. The relationship between microalbuminuria and ECG parameters were analyzed according to multiple regression and this was corrected by BP and pharmacological therapy (oral antidiabetic medication, diuretics, ACEI, ARB, aldosterone antagonists, calcium-channel blockers, beta-blockers, lipid lowering therapies, and antiplatelet agents). A *p* value <0.05 was considered significant. 

## 3. Results

A total of 109 patients (50 men; 67 ± 9.9 years) diagnosed with HTN who had had a digitally recorded ECG constituted the study group (Table 2). There were no differences in baseline characteristics or in BP between the groups. However, total and low-density-lipoprotein (LDL) cholesterol and were significantly higher in the normo–normo group vs the normo–micro group (188.1 ± 28.9 vs 163.2 ± 23.1; *p* = 0.001 and 109.5 ± 27.2 vs 85.5 ± 15.6; *p* < 0.001, respectively). Similarly, high-density-lipoprotein (HDL) cholesterol was significantly lower in the micro–micro group compared to the normo–normo group (51.0 ± 3.1 vs 43.1 ± 9.2; *p* = 0.001). No other differences in cholesterol levels were observed between the other groups.

There were no differences in medical therapies between groups (Table 3).

**Electrocardiogram**: The study groups showed no differences in the presence of LVH (diagnosed using the ECG platform considering the presence of at least one LVH criteria as indicative of LVH). When individual criteria were analyzed, RV6/RV5 >1 was observed more frequently as renal function declined: normo–normo (10.3%; 0.8 ± 0.2), normo–micro (15.8%; 0.8 ± 0.4), micro–micro (37.5%; 1.0 ± 0.5) (proportion of RV6/RV5 and RV6/RV5 value, respectively) (*p* = 0.025). 

The 12-lead QRS-complex voltage-duration product was significantly increased in the normo–micro group (1388.6 ± 419.0) compared with the other 2 groups (normo–normo: 1185 ± 261.1; micro–micro: 1101.9 ± 359.7) (*p* = 0.006), reflecting an increase in the QRS-complex voltage-duration product in patients without microalbuminuria at the start of the follow-up who then went on to develop microalbuminuria. Furthermore, the frontal plane QRS axis was more leftward in the groups with a higher degree of microalbuminuria: normo–normo (13.9 ± 29.5°), normo–micro (10.0 ± 34.4°), micro–micro (−11.1 ± 36.7°).

Patients with baseline microalbuminuria (*n* = 50) who presented LVH according to the ECG platform criteria (*n* = 35) showed a non-significant tendency of higher degree of microalbuminuria when compared with those who did not present LVH (*n* = 15): 42.7 ± 84.1 vs 26.2 ± 64.7, respectively; *p* = 0.053.

When analyzing patients who developed microalbuminuria during the follow-up (*n* = 51), those with positive Cornell voltage criteria (*n* = 12) presented a lesser degree of progression of microalbuminuria when compared with the rest of this subgroup (59.1 ± 51.4 vs 146.9 ± 444.8; *p* = 0.044, respectively). Nevertheless, no significant correlation between baseline microalbuminuria or its progression with any ECG criteria was observed, with only the Cornell voltage criteria showing a non-significant association with a lower degree of microalbuminuria (*r* = 0.18; *p* = 0.085). 

When BP levels were considered, analyzing those with a BP ≥140/90 mmHg (*n* = 39), the Cornell voltage criteria showed a non-significant tendency to present a lower degree of microalbuminuria (27.4 ± 28.2 vs 37.5 ± 92.6; *p* = 0.052, respectively). However, no such tendency was observed in patients with BP <140/90 mmHg. In addition, when BP levels were corrected for, no correlation between microalbuminuria, or its progression during follow-up, and the platform ECG criteria, could be found. 

With regards to microalbuminuria at baseline, patients treated with ARB and diuretics (*n* = 23) who presented positive Cornell voltage criteria showed a higher degree of microalbuminuria compared to those with a negative Cornell voltage criteria (24.8 ± 15.1 vs 6.4 ± 6.8; *p* = 0.018). When considering the progression of microalbuminuria during follow-up, those with positive Cornell voltage criteria showed slower progression when treated with diuretics (*n* = 35) (71.0 ± 50.1 vs 147.8 ± 492.8; *p* = 0.035) or ARB (*n* = 36) (67.0 ± 44.2 vs 143.5 ± 523.8; *p* = 0.016). On the other hand, a significant correlation (−0.347; *p* < 0.05) between the microalbuminuria progression and the T-wave angle in the frontal plane was observed in these patients, showing a leftward deviation of the T-wave as microalbuminuria worsened throughout follow-up. 

The oral antidiabetic drug group were the only pharmacological group that showed a significant correlation between baseline microalbuminuria and positive Cornell voltage criteria (*n* = 30; *r* = 0.533; *p* < 0.05). No correlation between microalbuminuria progression and the ECG platform parameters were observed when medical therapy was corrected for.

## 4. Discussion

The main findings of this study was the identification of some ECG parameters, which predict renal disease progression in patients with HTN, namely: (1) T-wave angle in the frontal plane was inversely correlated with the progression of microalbuminuria; (2) voltage-duration QRS-complex product of the 12-lead ECG was greater in patients who developed microalbuminuria during follow-up; (3) positive Cornell voltage criteria predicted lesser degree of microalbuminuria progression in patients on ARB. 

In patients with HTN, renal damage is associated with cardiovascular disease, independently of the patient’s cardiovascular risk profile [18]. Also, in patients with a greater degree of renal dysfunction ECG alterations, such as LVH, have been observed [19,20]. Sciarreta et al. [21] studied the relationship between microalbuminuria and ECG alterations in patients without cardiovascular disease in a large Italian cohort, observing that several ECG alterations (arrhythmias, intraventricular conduction disturbances, repolarization alterations, and leftward frontal plane axis deviation) were independently associated with renal dysfunction, which may be of value in the prognostic evaluation of such patients. Our group found certain ECG alterations (heart rate, QRS-complex duration, and QRS-T angle in the frontal plane) to be associated with plasma creatinine levels in elderly patients, which shows a close relationship between organ damage in HTN, probably secondary to a common pathophysiological process [22]. The potential use of the ECG to screen for patients who may present continuous renal deterioration despite optimal medical therapy has not previously been analyzed. 

Our study results confirm some previously published results, such as the association between ECG parameters and renal dysfunction in patients with HTN. The increase in the 12-lead QRS-complex voltage-duration, linked to renal dysfunction in our study, is a precursor to intraventricular conduction defect as previously reported [21]. Furthermore, our data suggests that some ECG parameters (positive Cornell voltage criteria) allow for the identification of patients with renal dysfunction who are likely to show less deterioration in renal function (with or without medical therapy), which may be of interest in clinical practice. 

The Cornell voltage criteria have been used as a diagnostic tool for LVH in several HTN studies, and have been shown to have a prognostic value [22]. In addition, antihypertensive medications, such as olmesartan, has been shown to delay development of LVH (assessed by the Cornell voltage criteria) and reduce microalbuminuria [23]. In concordance with the aforementioned findings, our observations, including left axis deviation in the frontal plane and posterior deviation in the horizontal plane, suggest that cardiac remodeling evaluated by the ECG not only correlates with renal dysfunction, but also with the progression of renal dysfunction in patients treated with ACEI or ARB. This suggest that the ECG, a non-invasive readily available marker, may allow us to identify which patients may be at higher risk of progression of renal function, and to take appropriate therapeutic measures. 

The current study has some limitations; the number of patients included is relatively small, although we considered it sufficient to study the relationship between ECG parameters and microalbuminuria progression. Nevertheless, this is an initial study, which needs validation in a larger cohort and in other patient populations. Furthermore, LVH was not confirmed by echocardiography, although the LVH ECG criteria are more easily implemented and show good diagnostic precision, and the ECG analysis was automatized in this study, the latter which may reduce errors associated with manual ECG analysis. 

In summary, in the current study, we have identified some ECG parameters which predict CKD in patients with HTN, and may allow for the identification of patients who are at risk of CKD progression despite optimal antihypertensive pharmacotherapy. 

## Figures and Tables

**Table 1:** ECG criteria for left ventricular hypertrophy (LVH).

Criteria	Formula	LVH Criteria
Sokolow–Lyon Voltaje (mV)	S (V1) + max (R (V5) or R (V6))	≥3.5 mV
Cornell Voltaje (mV)	R (aVL) + S (V3)	≥2.8 mV (M) ≥2.0 mV (F)
R6:R5	R (V6)/R (V5)	>1
RaVL (mV)	R (aVL)	>1.1 mV
Gubner-Ungerleider (mV)	R (I) + S (III)	>2.5 mV
Lewis (mV)	(R (I) + S (III)) − (R (III) + S (I))	>1.7 mV
QRS 12 (mV)	R wave plus S wave (or Q wave, the largest) in all 12 leads	>19.530 mV (M) >18.499 mV (F)
HES	Logistic regression equation	
PDV Sokolow (msxmV)	S (V1) + max (R (V5), R (V6)) × QRS duration	>367.4 mv.ms (M) >322.4 mv.ms (F)
PDV Cornell	Males: R (aVL) + S (V3) × QRS duration Females: (R-aVL + S-V3 + 0.6 mv) × QRS duration	>244 mv.ms
PDV Gubner	Gubner × QRS duration	>207 mv.ms
PDV RaVL	RaVL × QRS duration	>103 mv.ms
PDV QRS 12	QRS area in 12 leads	>2348.8 mv.ms (M) >1960.7 mv.ms (F)
Dalfó	R (aVL) + S (V3)	>1.6 mv (M) >1.4 mv (F)
Perugia	(a) SV3 + RavL > 2.4 mV male or > 2.0 mV females, or (b) Left ventricular strain pattern, or (c) Romhilt–Estes point score ≥5	Any of them
Romhilt–Estes (points)		>4 or >5 points

**Table 2:** Baseline patient characteristics.

	Total (*n* = 101)	Normo–Normo (*n* = 51)	Normo–Micro (*n* = 29)	Micro–Micro (*n* = 21)	*p*
Age (years)	67.0 ± 9.9	65.7 ± 10.2	69.2 ± 8.1	66.9 ± 11.2	0.31
Men (%)	49.5	37.3	62.1	61.9	0.05
DM (%)	40.6	31.4	48.3	52.4	0.13
Smoker (%)	9.9	9.8	10.3	9.5	0.82
SBP (mmHg)	133.1 ± 14.5	133.2 ± 12.4	134.6 ± 16.9	130.6 ± 15.8	0.64
DBP (mmHg)	78.9 ± 10.6	79.5 ± 11.2	78.1 ± 11.3	78.4 ± 8.5	0.83
TC (mg/dL)	177.5 ± 30.1	188.1 ± 28.9	163.2 ± 23.1	171.3 ± 32.7	0.001 *
Triglyceride (mg/dL)	129.1 ± 62.7	124.2 ± 55.8	127.7 ± 58.3	143.1 ± 82.7	0.33
HDL (mg/dL)	51.0 ± 13.1	54.5 ± 13.4	50.5 ± 12.7	43.1 ± 9.2	0.003 **
LDL (mg/dL)	101.3 ± 26.0	109.5 ± 27.2	85.5 ± 15.6	101.5 ± 25.4	0.000 *
Creatinine (mg/dL)	1.0 ± 0.4	0.9 ± 0.2	1.1 ± 0.4	1.2 ± 0.5	0.000 ***
LVH (%)	63.4	56.9	65.5	76.2	0.29

* normo–normo vs normo–micro; ** normo–normo vs micro–micro; *** normo–normo vs normo–micro and normo–normo vs micro–micro; DBP = diastolic blood pressure; DM = diabetes mellitus type 2; HDL = high-density lipoprotein; LDL = low-density lipoprotein; LVH = left ventricular hypertrophy (at least one component of the ECG LVH algorithm present); SBP = systolic blood pressure; TC = total cholesterol.

**Table 3:** Medical therapy.

Medical Therapy	Total (*n* = 101)	Normo–Normo (*n* = 51)	Normo–Micro (*n* = 29)	Micro–Micro (*n* = 21)
DT (%)	30.7	25.5	31.0	42.9
Diuretics (%)	37.6	25.5	51.7	47.6
ACEI (%)	14.9	11.8	13.8	23.8
ARB (%)	40.6	37.3	41.4	47.6
CA (%)	49.5	52.9	41.4	52.4
BB (%)	25.7	27.5	24.1	23.8
AB (%)	20.8	15.7	31.0	19.0
CA + ARB (%)	13.9	9.8	13.8	23.8
ARA + DIU	22.8	33.3	20.7	0.0
Antiplatelet	36.6	37.3	44.8	23.8
Statin	73.3	74.5	72.4	71.4
AA	10.9	15.7	10.3	0.0

AA = aldosterone blocker; AB = alpha-blockers; ACEI = angiotensin converting enzyme inhibitor; ARB = angiotensin 2 receptor blockers; BB = beta-blockers; CA = calcium antagonists; DT = diabetic treatment.
